# *Bactericera tremblayi* (Wagner, 1961) (Hemiptera: Triozidae): The Prevalent Psyllid Species in Leek Fields of Northwestern Spain

**DOI:** 10.3390/insects15010004

**Published:** 2023-12-21

**Authors:** Yolanda Santiago-Calvo, Laura Baños-Picón, Diego Flores-Pérez, M. Carmen Asensio-S.-Manzanera

**Affiliations:** 1 Instituto Tecnológico Agrario de Castilla y León (ITACyL), Ctra. de Burgos Km. 119, 47071 Valladolid, Spain; sancalyo@itacyl.es (Y.S.-C.); floperdi@itacyl.es (D.F.-P.); 2 Departamento de Biología Animal, Facultad de Biología, Universidad de Salamanca, 37007 Salamanca, Spain; lbanos@usal.es

**Keywords:** *Bactericera nigricornis* Förster complex, *Bactericera nigricornis*, *Bactericera trigonica*, sweep net, horizontal water traps, developmental time, survival

## Abstract

**Simple Summary:**

The cultivation of leek in Castile and Leon is located in the provinces of Segovia and Valladolid, which accounts for the largest production of this crop in Spain. In 2015, growers became concerned by severe damage to leek crops, tentatively attributed to the onion and leek psyllid, *Bactericera tremblayi*. For effective integrated pest management in leek fields, it is essential to understand the biology of this species and the factors influencing its development. This study revealed that *B. tremblayi* predominated as the primary species of jumping plant-lice in leek crops throughout the entire crop cycle, from May–July to harvest (September–November). The maximum peaks of *B. tremblayi* were observed at the end of the crop cycle, particularly during late-season cycles characterized by lower mean temperatures. Under controlled conditions, *B. tremblayi* exhibited its complete development from the egg stage to adult within the temperature range of 15 to 25 °C. Notably, when the temperature exceeded the threshold of 30 °C, *B. tremblayi* did not complete its developmental cycle. The optimum temperature for the development of *B. tremblayi* was close to 24 °C. The findings of this study significantly contribute to the development of rational population management strategies for *B. tremblayi*. This knowledge plays a crucial role in mitigating the potential impact of this species on agricultural systems.

**Abstract:**

*Bactericera tremblayi* (Wagner, 1961) (Hemiptera: Triozidae), the onion and leek psyllid, belongs to the *Bactericera nigricornis* Förster complex, along with *B. trigonica* and *B. nigricornis*. In contrast to the other two species, there has been a notable absence of studies examining the distribution and seasonal occurrence of *B. tremblayi*, despite its association with significant issues in leek crops. Surveys were conducted between 2017 and 2020 in the main leek-growing area of Castile and Leon (Spain). An extensive survey encompassing 29 distinct plots was monitored with sweep nets and visual inspection, counting plants with immature forms at three times in the crop cycle. Additionally, a total of seven seasonal monitoring surveys were conducted in the same area of study. Plots were monitored every ten days, employing three distinct sampling methods including horizontal green tile water traps, sweep nets, and visual inspection, counting the juvenile stages by plant. The results revealed that *B. tremblayi* predominated as the primary species of jumping plant-lice in leek crops throughout the entire crop cycle. To date, there exists no documented incidence of pathogenic agents within symptomatic leeks. Consequently, the manifestation of severe symptoms is highly likely to be a direct consequence of the feeding activity of the onion psyllid. Populations of *B. tremblayi* were present in leek crops from May–July to harvest (September–November). Adults were captured in horizontal green water traps several days before they were found in sweep net samples, making the former effective in capturing early immigrant individuals. The maximum peaks of *B. tremblayi* were observed at the end of the crop cycle, particularly during late-season cycles characterized by lower mean temperatures. During observations made in a controlled environment, temperature exerted a significant influence on the developmental time of all stages of *B. tremblayi*. The complete development from egg to adult occurred within a temperature range of 15 to 25 °C. At 30 °C, the survival of eggs and N1 nymphs was limited and *B. tremblayi* did not complete its developmental cycle. The optimum temperature for the development of *B. tremblayi* provided by the models used was close to 24 °C with the application of Briere, Taylor, and Lactin models and around 21 °C with the SSI model. These results provided a good adjustment in predicting the survival patterns of *B. tremblayi* under the studied environmental conditions.

## 1. Introduction

The leek (*Allium ampeloprasum* var. *porrum* (L.) J. Gay) is a minor extensive horticultural crop in Spain, characterized by a comparatively limited cultivated area compared to other horticultural products such as potatoes and tomatoes. Despite this, it has significant economic importance, and its production is concentrated in the regions of Castile and Leon and Andalusia. The cultivation of leek in Castile and Leon, with 862 ha [1], is located in central Spain, specifically in the provinces of Segovia and Valladolid, which accounts for the largest production of this crop in Spain. Owing to its economic relevance, there is a lack of knowledge and studies conducted on the pests affecting this crop.

In 2015, growers became concerned by severe damage to leek crops [2]. The observed symptoms included the presence of yellow stripes on the cylinder, potential bursting of the bundled leaf sheaths, and the emergence of roots between the ruptured sheaths. Concurrently, the aerial tips of the green leaves exhibited withering, resulting in a change in colour from bluish green to dark shiny green, ultimately leading to potential plant mortality. These symptoms had been previously reported and described by Ouvrard and Burckhardt (2012) [3] in different agricultural leek crop areas in France in 2000, showing gradual degrees of damage depending on the specific area and year. The damage was associated with high populations of the onion and leek psyllid in both the aforementioned cases. 

*Bactericera tremblayi* (Wagner, 1961) (Hemiptera: Triozidae), the onion and leek psyllid, belongs to the *Bactericera nigricornis* Förster complex [4], which also includes *B. trigonica* (Hokinson, 1981) and *B. nigricornis* (Förster, 1848). This group is composed of three multivoltine species [5] that exhibit morphological similarities, have a diverse range of herbaceous plants as hosts and overwinter as adults [3]. *Bactericera tremblayi* exhibits a wide distribution in the Mediterranean region, encompassing Spain (both the mainland and the Canary Islands), France, Greece, Turkey, and Italy. Furthermore, there have been documented occurrences of this species in other countries such as Serbia, Bulgaria, Jordan, and Iran [6]. Species of the genus *Allium* [5] and the families Brassicaeae [7], Apiaceae [8], and Caryophyllaceae [7] has been cited as hosts of *B. tremblayi*.

*Bactericera trigonica* and *B. nigricornis* present overlapping areas of distribution with *B. tremblayi* as well as polyphagous habits and both have been reported in Spanish horticultural production areas [9,10,11]. *Bactericera trigonica* feeds mainly on carrots and celery, among other members of the Apiaceae family [4], and is responsible for the transmission of the phloem-restricted bacterium *Candidatus* Liberibacter solanacearum (*Ca*. L. solanacearum) causing several damages in these crops in Spain [9,12]. *B. nigricornis* has been reported on wild weed species belonging to the families Amaranthaceae, Boraginaceae, Brassicaceae, Liliaceae, Papaveraceae, and Solanaceae [4] and on different cultivated plants such as carrot, parsley, potato, beets, brassicas, and radish [4,7]. Additionally, *B. nigricornis* has been reported on both carrot and potato [10,11] but not on leek [13]. Under controlled conditions, Antolínez et al. in 2017 [14] reported that *B. tremblayi* fed on carrot and potato and acquired *Ca*. L. solanacearum, although it was unable to transmit the bacterium. Furthermore, no occurrences of *Ca.* L. solanacearum or other known pathogens have been detected in symptomatic leek crops so far.

Despite *B. tremblayi* has been associated with important damages on leek crops in countries where its presence has been reported, investigations that focus specifically on this species are remarkably scarce. However, *B. trigonica* and *B. nigricornis*, the other two species of the complex, have been extensively studied in terms of their distribution and seasonal occurrence. Ouvrard and Burckhardt (2012) [3] have emphasized the necessity for further comprehensive research involving field monitoring and extensive entomological surveys. Such investigations are imperative to establish a conclusive correlation between the observed symptoms and the damage caused by *B. tremblayi*, as well as to elucidate the precise nature of this interaction. 

In addition to studying the population dynamics of *B. tremblayi* in leek fields, it is essential to provide information regarding its biology and the factors that influence its development. The rate of insect development and survival is profoundly impacted by the ambient temperature, which stands out as the most crucial climatic factor influencing these organisms [15]. Evaluating the relationship between insect development and temperature proves valuable for anticipating the seasonal occurrence and population dynamics of insect species. Moreover, an insect’s ability to undergo development at varying temperature ranges represents a vital adaptation enabling survival under diverse climatic conditions [16]. The relationship between temperature and development time in insects is widely studied and has been described using various models, which are most often used to predict the activity and seasonal population dynamics of pests in field conditions and are useful in making pest management decisions [17].

Considering the economic significance of leek cultivation in the region of Castile and Leon and the potential crop losses to psyllid damages, there is a compelling need for further research and investigations. To this end, our specific objectives were: i, to identify the psyllid species associated with leek crops in the main producing area in Spain, specifically within the region of Castile and Leon; ii, to investigate the seasonal occurrence and abundance patterns of *B. tremblayi* and species belonging to the *B. nigricornis* Förster complex; iii, to evaluate the developmental rate and survival of *B. tremblayi* at four constant temperatures; iv, to estimate the temperature thresholds and thermal requirements of this species. 

To achieve these objectives, extensive and seasonal surveys were executed. In parallel, a colony of *B. tremblayi* was studied to monitor the developmental progression under specified fixed temperatures. The outcomes of this research will provide valuable insights into the seasonality and abundance of *B. tremblayi* in the investigated area and into its survival and development as a function of temperature.

## 2. Materials and Methods

### 2.1. Study Area and Sampling Design 

An extensive survey encompassing 29 distinct plots was conducted in the main leek cultivation region of Castile and Leon located in Valladolid and Segovia provinces during the cropping seasons from 2017 through 2020 (Table S1). The classification of these fields was based on their crop cycle, distinguishing them into two categories: mid-season (MS) fields, transplanted by the month of May and early June, and late-season (LS) fields, transplanted by late June. The observations were carried out at three critical junctures: at 6 weeks after transplantation, at the midpoint of the crop cycle, and at the harvest phase. The monitoring of adult psyllids was conducted by employing the sweep net sampling method. The assessment of immature forms was realized by evaluating the presence of eggs and/or nymphs in one thousand randomized plants in the plot. 

Additionally, a total of seven seasonal monitoring surveys were conducted in the same area of study between 2017 and 2020 (Table 1). These surveys occurred in two leek fields per year from 2017 to 2019 and a single field in 2020. The plots, which were at least one hectare in size, were monitored every ten days during the main season of the crop, from the months of May–June–July (transplanting) to September–October–November (crop harvesting). The crop management in leek plots may be to either integrated or organic. Integrated agriculture is characterized by the use of insecticides authorized by the Register of Products of the MAPA [18], in accordance with the Integrated Guide for controlling pests in the Liliaceae family [19]. In the context of organic production, the application of insecticides is subject to regulation by the European Commission [20]. Three different sampling methods were used: horizontal green tile water traps, sweep nets, and visual inspection, counting juvenile stages per plant. The daily mean temperature data were acquired from the nearest meteorological stations (Olmedo in Valladolid and Gomezserracín in Segovia). 

### 2.2. Sampling Methodology 

Leek canopies were sampled using a telescopic folding sweep net to capture adult psyllids in extensive and seasonal monitoring surveys. A total of ten independent samples were randomly taken from each plot on the sampling dates. Each sample consisted of ten consecutive sweeps that were placed in a plastic zip-bag and stored in the laboratory at -20 °C for 48 h to kill the insects. Then, samples were preserved in 70% ethanol and stored in 1.5 mL vials for sorting by gender and identification of psyllid species [10]. The identification process was carried out by conducting a morphological examination following the description of the species of the *Bactericera nigricornis* Förster complex [3,5] using a Nikon SMZ1000 Zoom Stereo Microscope (Melville, NY 11747-3064, USA), based on male and female genitalia. To determine the presence of juvenile stages, such as eggs or nymphs, in extensive surveys, visual inspection was carried out by counting the number of plants containing immature psyllids. For each date and field location, one hundred randomly selected plants were examined. 

Seasonal leek plots were evaluated every ten days. The capture of adult psyllids was conducted through the utilization of two methodologies. Firstly, sweep nets were used as described above. Secondly, a horizontal green tile water trap, with slight modifications [10], was employed, owing to their neutral nature. This trap, also known as an Irwin trap, was explicitly designed to capture alate insects [21,22]. Positioned at the canopy level within a designated quadrant of the plot measuring 10 m × 10 m and left untreated with insecticide, the trap consisted of a methacrylate container (16.5 × 16.5 × 4.5 cm) housing a square ceramic tile (15.5 × 15.5 cm). The container was filled with a 50% solution of ethylene glycol in water. The trap content was filtered using a funnel and the collected insects were preserved in 70% ethanol until taxonomic identification could determine that they belonged to the *Bactericera nigricornis* Förster complex and separated by gender [3,4]. Additionally, twenty randomly selected leek plants were visually inspected to assess the presence of immature psyllids, counting the number of eggs and nymphs in developmental stages N1–N2 and N3–N5 on each plant. An index value ranging from 0 to 4 was utilized to estimate the relative abundance of eggs and nymphs, where 0 represented no presence, 1 indicated 1 to 4 individuals, 2 encompassed 5 to 20 individuals, 3 indicated 21 to 50 individuals, and 4 represented more than 50 eggs or nymphs. 

In order to identify the psyllid species developing in the leek crops and establish the colony for use in assays under controlled conditions, a mean of 20 nymphs N4–N5 were collected from three fields. These juvenile forms were subsequently reared in the laboratory in individual cages at 18–22 °C, and the emerged adults were identified. Only the species *B. tremblayi* was present among the reared specimens. A population of onion psyllids was established and maintained on leek plants in pots in two mesh rearing cages (dimensions: 47.5 × 47.5 × 93.0 cm) in a greenhouse with a medium temperature of 22 °C, 50% humidity, and a photoperiod of 16:8 h (L:D). 

### 2.3. Development and Survival of Juvenile Stages at Different Constant Temperatures

Leek plants (cultivar Longton) with at least four leaves were infested with 1–5-day-old adult psyllids. After oviposition for 24 h, plants with more than 50 eggs were selected for the assay. 

Laboratory experiments were conducted using an environmental chamber (MLR 350H, SANYO Electric Company) set at a constant relative humidity (65 ± 5%) and photoperiod (16:8 L:D). The response to temperature was assessed by exposing *B. tremblayi* eggs to four constant temperatures (15, 20, 25, 30 °C), in separate experiments, allowing the eggs to develop into adults. The experiment, consisting of five infested leek plants placed in individual cages, was conducted in triplicate for each temperature, each time with eggs from different cohorts. Daily recording of the number of individuals in each immature stage was conducted to determine the hatching rates and development time at different temperatures. 

### 2.4. Thermal Models and Critical Temperatures Estimation 

The reciprocal of developmental time for different stages of *B. tremblayi* was calculated to obtain the developmental rate. The degree day model [15] was used to estimate the linear relationship between temperature and the rate of development of total immature stages as well as the lower temperature limit for development of *B. tremblayi*. To describe the developmental rate over a wider temperature range, three of the most common non-linear models that have been developed, including Briere-1, Taylor, and Lactin-2 [23,24,25], were used. In addition, the Sharpe–Schoolfield–Ikemoto (SSI) model based on theoretical studies of enzyme thermodynamics was used [26]. 

Regarding critical temperatures, the minimum temperature (Tmin), maximum temperature (Tmax), and optimum temperature (Topt) were estimated using the above-mentioned models [27]. Tmin and Tmax represent the tolerance limits for insect development, while Topt is the temperature at which the development rate is maximum. Finally, the Sharpe–Schoolfield–Ikemoto (SSI) complex model enabled us to estimate the intrinsic optimum temperature at which the population size is maximal with the lowest mortality (Topt’), the temperature at which the enzyme is half active and half low-temperature inactive (TL), and the temperature at which the enzyme is half active and half high-temperature inactive (TH) [28,29,30]. 

### 2.5. Data Analysis 

The Shapiro–Wilk normality test and Levene homoscedasticity test were performed prior to data analysis. 

Differences between the types of crop cycle in the extensive survey were tested with a one-way analysis of variance using the statistical software R version 4.2.3. (R Development Core Team, 2023). Homoscedasticity was confirmed for the two factors studied. The separation of means between the number of insects found by sweeping and number of plants with immature forms by type of crop cycle was subjected to a nonparametric Kruskal–Wallis test (*p* < 0.05). 

The effect of temperature on the developmental period and survival of *B. tremblayi* was analyzed with a one-way analysis of variance (ANOVA) of the trial means and the means by temperature were separated using Tukey’s Honestly Significant Difference (HSD) multiple comparison (*p* < 0.05) for each immature form using the R program. 

Statistical analyses referring to model adjustment were carried out using the ‘devRate’ R package with four models, including Campbell, Briere-1, Taylor, and Lactin-2. The OptimSSI program (version 2.7), which runs on the R statistical software, was used to estimate the thermodynamic model parameters in the SSI model [30].

## 3. Results

### 3.1. Extensive Survey

**Figure 1:**
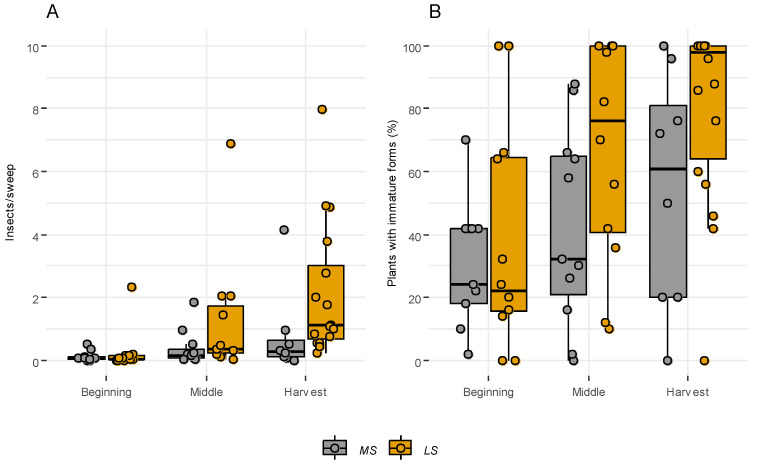
Number of adults by sweep (**A**) and percentage of plants with immature forms (**B**) of *Bactericera tremblayi* collected by crop cycle (MS: mid-season, LS: late-season) at the beginning and middle of the crop cycle and at harvest in leek plots subject to extensive survey in Castile and Leon (Spain) from 2017 to 2019.

The initial surveys were conducted in May–June and July–August in mid-season and late-season plots, respectively. The recorded captured adult psyllids were notably low across both crop cycles, and the percentage of plants with immature forms exhibited considerable variability, ranging from 0% to 100% (Table S1). However, no significant differences were observed between crop cycles (Figure 1).

Subsequent monitoring of mid-season leek plots occurred in July–August, while late-season plots were observed from late August–September. Significant differences were observed in the number of captured insects (Kruskal–Wallis chi-squared = 6.151, df = 1, *p*-value = 0.013) and the percentage of plants with juvenile stages (Kruskal–Wallis chi-squared = 3.898, df = 1, *p*-value = 0.048) between mid-season and late-season plots (Figure 1).

The maximum captures and the highest percentage of plants with immature psyllid stages were observed towards the end of the crop cycle, closer to the harvest period (Table S1). Monitoring for mid-season and late-season leek plots occurred in late August–September and October–February, respectively. During this period, discernible differences in captures were evident (Kruskal–Wallis chi-squared = 5.6013, df = 1, *p*-value = 0.018), and also in the number of plants with immature forms (Kruskal–Wallis chi-squared = 6.553, df = 1, *p*-value = 0.010) (Figure 1). 

### 3.2. Seasonal Monitoring 

**Figure 2:**
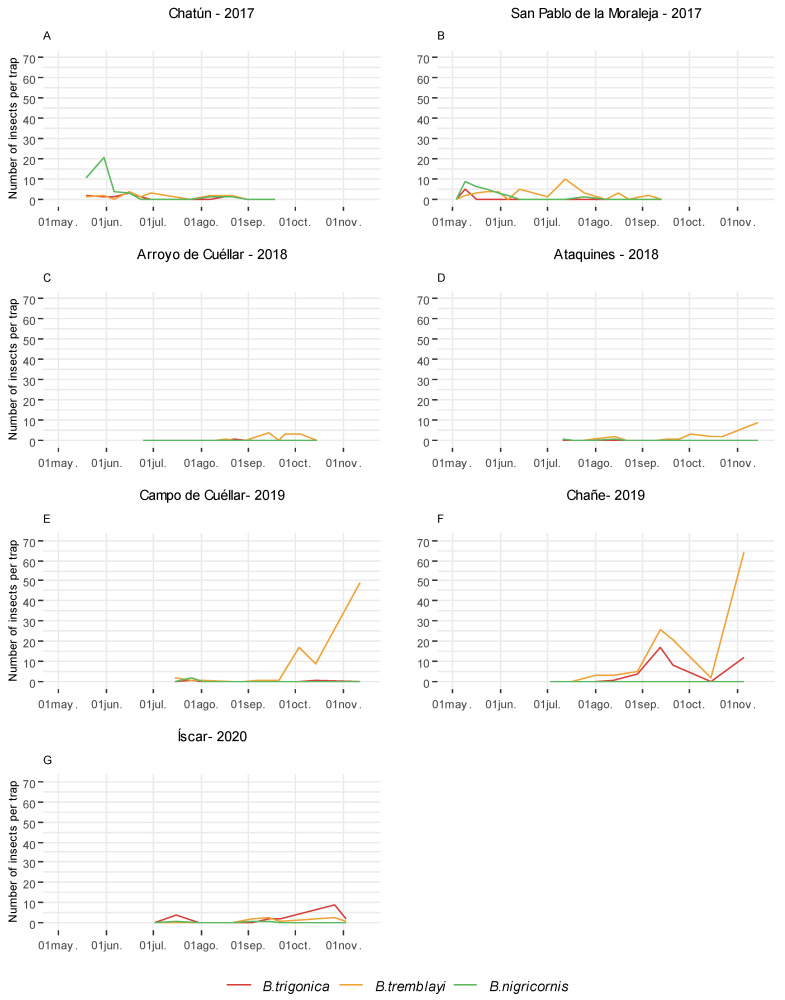
Number of psyllids of *Bactericera nigricornis* Förster group (*B. trigonica*, *B. tremblayi*, and *B. nigricornis*) collected in leek plots subject to seasonal monitoring in Castile and Leon (Spain) using horizontal green water traps from 2017 to 2020.

*Bactericera tremblayi* emerged as the dominant species in terms of abundance when subjected to horizontal green tile water trap and sweep net sampling across all surveyed plots throughout the observation period (Table 2). The presence of *B. trigonica* and *B. nigricornis* was minimal in sweep nets, with considerably fewer specimens compared to those acquired via horizontal green water traps (Table 2). It is noteworthy to highlight the appearance of these two species at specific time points in horizontal green water traps (Figure 2). In 2017 at Chatún, the population of *B. nigricornis* exceeded that of the other species, emerging as the most abundant during the month of May (Figure 2A). Conversely, the presence of *B. trigonica* is remarkable, particularly during the months of September and November at Chañe in 2019 (Figure 2F) and at Íscar in 2020 (Figure 2G), where it displayed the highest prevalence and garnered special consideration in the latter plot.

**Figure 3:**
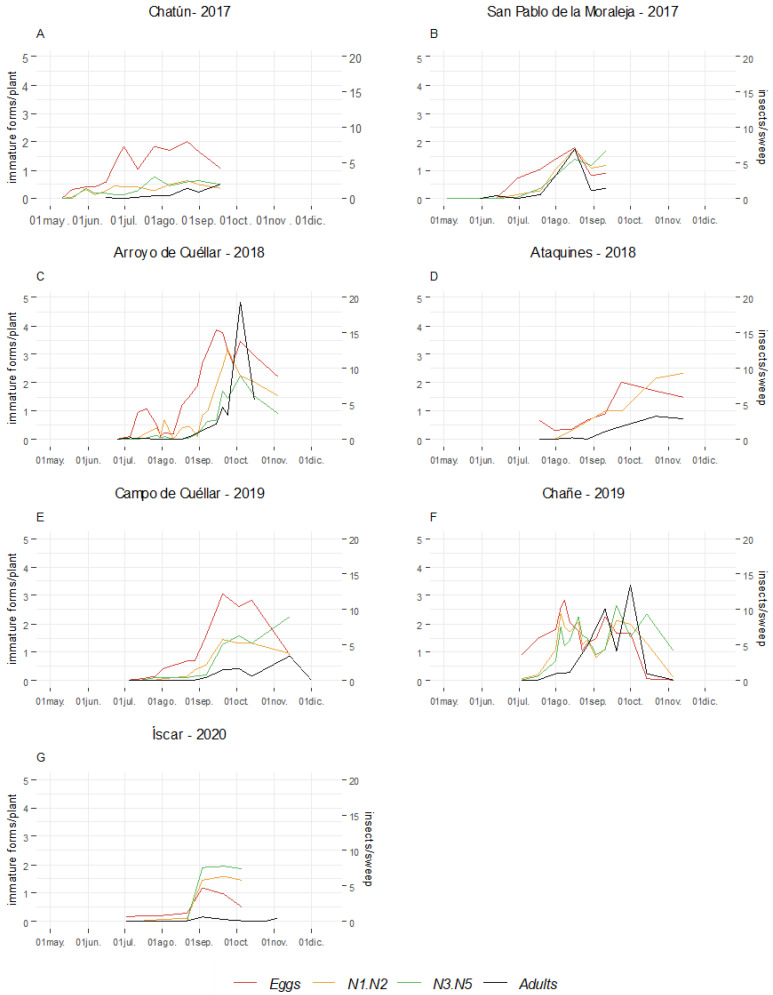
Number of eggs, nymphs N1–N2, and nymphs N3–N5 found on plants in leek plots subject to seasonal monitoring in Castile and Leon (Spain) from 2017 to 2020. Scale used 0 = 0, 1 = 1–4, 2 = 5–20, 3 = 21–50, 4 = more than 50 (first y-axis). Number of individuals of *Bactericera tremblayi* by sweep (second y-axis).

Regarding the seasonal abundance of captures of *B. tremblayi* from sweep net samplings, it was observed that both monitored plots in 2017, with mid-season cycles, and the plot monitored in 2020 exhibited consistently low capture counts throughout the entire cycle (Figure 3). Between 2018 and 2019, with late-season cycles in the surveyed plots, the capture counts were higher, especially in 2019 (Figure 3). The emergence of adult onion psyllids in leek crops was noted during the months of May and June. This observation was made in the context of mid-season crops in the year 2017. Subsequently, the population exhibited a gradual increase, reaching a peak occurrence in August. Nevertheless, starting in September, the populations showed an upward trend in late-season crops, reaching its maximum peaks in October.

Analysis of the gender distribution among the captured adults of the three species revealed a higher prevalence of males. 

The temporal dynamics of *B. tremblayi* immature forms exhibited a sequential pattern of peaks for eggs, N1–N2 nymphs, and N3–N5 nymphs, depending on the field plots and prevailing climatic conditions (Figure 3). Generally, the observed egg counts were higher compared to the rest of the subsequent immature stages. In several plots, the maximum levels of N3–N5 nymphs were observed at the same time as the peaks of the next adult population, suggesting a growing population trend. 

In 2017, eggs and nymphs were observed from early June until the end of the crop cycle (Figure 3A,B). During the growing seasons of 2018, 2019, and 2020, in the context of late-season crops, values index for eggs and nymphs were evident from July, with relative peaks observed during August and September. However, the maximum peaks of N1–N2 and N3–N5 nymphs occurred from late September to November.

### 3.3. Developmental Time and Survival of Immature Stages of B. tremblayi 

The developmental time was significantly influenced by temperature for all stages of *B. tremblayi* (Table 3). The complete development from egg to adult took place within the temperature range from 15 to 25 °C, with shorter developmental durations observed at higher temperatures. Particularly, the shortest developmental period of 26.72 ± 2.78 days was recorded at 25 °C. 

Regarding survival rates, temperature significantly affected the eggs’ and N1–N2 nymphs’ development, with significant differences observed between 15 °C and 30 °C (Table 4). At 30 °C, limited egg and N1 nymph survival was observed, with a survival percentage of approximately 8%. Conversely, no statistically significant differences in survival were observed for the remaining nymphal stages at the temperatures within which *B. tremblayi* successfully completed its development. Survival rates for these stages ranged between 82.12 ± 13.3% and 96.73 ± 2.04%. 

The emergence of females was observed to be significantly higher than that of males, with proportions of 53.15% and 46.85% (*p* = 0.03), respectively. However, the temperature did not influence the sex ratio or the relative development time of males and females.

### 3.4. Model Evaluation

The development rate for eggs and N1 nymphs of *B. tremblayi* exhibited a linear increase within the studied temperature range of 15 to 30 °C. However, the developmental rate for N2 nymphs throughout the entire life cycle also showed a response adjusted to a linear regression model, but only within the temperature range of 15–25 °C. 

With the exception of Campbell’s linear model, the results estimated using the other nonlinear models indicated that the thermal requirements for egg hatching were lower than those for nymphal stages. Specifically, the minimum temperature required for egg development was lower, while the maximum temperature required for egg development was higher than those required for nymphal stages (Table 5). Despite the models achieving high R^2^ adj values, the temperatures estimated with the Brière, Taylor, and Lactin models did not align with the temperatures observed during the development of *B. tremblayi* in the study area.

The Briere, Taylor, and Lactin models displayed comparable results for both maximum and optimal temperatures required for the nymphs. N1 nymphs exhibited the highest values for optimum and maximum temperatures, with values near 29 °C and 37.25 °C, respectively. Throughout the rest of the developmental stages, the values of temperatures exhibited notable similarity. The identified optimal temperature range varied from 23.70 °C to 25.76 °C, while the maximum temperatures spanned from 28.49 °C to 30.87 °C (Table 5). 

The SSI model was the only one that behaved differently compared to the other models, with a lower range of temperatures for all immature stages. The optimum temperature estimated using this model (Topt’) ranged between 20.54 and 21.93 °C, while the high temperature conditioning the development was between 25.83 and 37.18 °C for the different developmental stages. 

## 4. Discussion

In contrast to the other two species belonging to the *Bactericera nigricornis* complex [4], there has been a notable absence of research on the seasonal occurrence of *B. tremblayi.* This is despite the fact that the presence of the onion psyllid and its associated severe symptoms, as described by Ouvrard and Burckhardt (2012) [3], has been reported in leek crop areas including France [3], Spain [2], and Iran [31]. In our study, *B. tremblayi* was consistently observed in leek crops as the predominant species of jumping plant-lice throughout the entire crop cycle, as ascertained through the utilization of various sampling techniques in extensive and seasonal surveys. Among the three psyllid species examined belonging to the *B. nigricornis* group, *B. tremblayi* stands out as the sole species with the capacity for oviposition and successful completion of its life cycle on leek plants. As no known pathogen has been reported in symptomatic leeks to date, *B. tremblayi* seems to be the primary agent responsible for the damage observed in leek crops, a consequence of psyllid feeding. 

In our study, *B. nigricornis* specimens were exclusively detected in horizontal green water traps during the month of May in mid-season-cycle leek fields in 2017. The presence of this species in the aforementioned period can be attributed to its proximity to a harvested Brassicaceae crop, the host plant of *B. nigricornis*. In addition, *B. trigonica* was observed in horizontal green water traps from the month of August. Notably, in this horticultural production area, leek and carrot crops share cultivation cycles and spatial adjacency, which may facilitate the migration of insect populations between these crops. Since the month of August, early cycles of carrot crops are typically harvested, and during this period, there is a notable movement of psyllids from carrot plants to leek crops, presumably in search of new plants to colonize. To date, there is a notable dearth of research about the influence of harvest-induced resource depletion on the population dynamics and migratory behaviours of pest psyllids associated with cultivated plant species. Nevertheless, it is crucial to recognize that agricultural crops represent an immediate and concentrated source of predictable resources for local insect pests [32]. Particularly in the context of annual crops, such as vegetables, the crop cycle is relatively short, and the complete exhaustion of resources during harvest necessitates the dispersal of the pest population, leading to spatial movements [33]. In this scenario, the presence of these polyphagous psyllid species assumes significance, as they are capable of sequentially exploiting various host plants within a single growing season [34]. 

*Bactericera tremblayi* was the most frequently observed species in sweep nets in leek crops during the entire crop cycle in both the extensive survey and seasonal monitoring. However, the occurrence of *B. trigonica* and *B. nigricornis* was observed to be quite scarce during the sweep net sampling in both surveys. It is worth noting that sweep net sampling, when performed at the crop canopy level, has been demonstrated in previous studies to provide an accurate estimation of the psyllid species present on crops [10,11]. Conversely, sweep net sampling proved to be a valuable method for measuring psyllid populations across the entire leek cultivation cycle. This method has been widely used to obtain relative estimates of other psyllid species populations such as *Bactericera cockerelli* (Šulc, 1909) [35]. Instead, the number of onion psyllids collected during the leek cultivation cycle in horizontal water traps was relatively low when compared with the specimens captured with sweep net. However, adult psyllids were captured in horizontal green water traps several days prior to their detection in sweep net samples. These observations are in accordance with Láska (2013) [36], who indicated that water traps were suitable for capturing early immigrant psyllids. The maximum population movements of onion psyllids were detected using water traps around November, which correlated with their migration due to harvest. The detection of *B. tremblayi* populations using water traps before their observation in sweep net samples underscores the efficacy of water traps for early monitoring and identifying immigration events. The combination of these two sampling methods provided complementary data, proving to be valuable in detecting the presence of these psyllid species within the leek crop. 

Populations of *B. tremblayi* were observed in leek crops from May, persisting until the harvest period spanning from September to November, as observed in both the extensive survey and seasonal monitoring. In the context of seasonal monitoring, an increase in population was observed in sweep net sampling until mid-September, which coincides with the harvest period of the mid-season crop cycles. This increase in population density during the summer months, coupled with higher captures than those observed in our study, has been observed in other species, such as *B. trigonica* within the same surveyed area [10] and *B. cockerelli* in potato fields in northwestern New Mexico [37]. Despite this, *B. tremblayi* populations displayed noteworthy fluctuations, with the occurrence of one or two additional peaks characterized by higher values. These peaks were specifically observed in the late-season crop cycles, encompassing the period from September to the time of late harvests in November. Coincidentally, a similar population increase was also documented by Antolínez et al. [10] in *B. trigonica*, in which high population were recorded in early October in plots cultivated as late-season carrot fields. 

The impact of high temperatures with low humidity, common weather conditions observed during the summer in the Mediterranean area, is known to exert a significant influence on the life cycle completion of several psyllid species, leading to reduced fecundity, increased mortality, and slower rates of development at temperatures above the optimum [5]. The growth of *B. tremblayi* populations in field exhibited a distinct correlation with periods of lower daily air temperatures in our study. In the context of mid-season crops, it is noteworthy that the highest population peaks of *B. tremblayi* were documented during periods characterized by temperatures within the range of 20 °C to 25 °C (Figures S1 and S2). Conversely, in late-season crops the maximal surges in *B. tremblayi* populations typically occurred in the late stages of September or early October, coinciding with the period when mean temperatures hover around 15 °C. These results are consistent with those obtained from the developmental assays conducted under controlled conditions. The findings demonstrate that elevated temperatures correspond to accelerated developmental times but also result in a decline in survival rates for the species. Notably, a critical threshold for survival was observed at 30 °C, where both eggs and N1 nymphs exhibited a survival rate of only 8%. Moreover, the insect was unable to complete its life cycle at this critical temperature threshold of 30 °C, which is commonly prevalent during the summer season in the study area. Similarly, studies on species such as *B. cockerelli* have shown that at 31 °C under controlled conditions, although it was capable of completing its life cycle, the survival percentage was only 7% on tomato and 11% on potato [38]. 

Field observations revealed that egg values were notably higher than those of nymphs, suggesting mortality rates higher in younger immature stages. This fact is consistent with the results of assays conducted under controlled conditions where the maximum egg hatching was 56%. Studies conducted on *B. cockerelli* also reported higher mortality rates during the earlier stages, yet with relatively lower values than those observed in *B. tremblayi*, with survival rates ranging from 63 to 85% [38,39,40]. It is worth noting that, under field conditions, egg hatching exhibits lower rates, attributed to environmental factors such as strong winds, rainfall, cultural practices, and predation by beneficial arthropods or birds. In a study conducted by Yang et al. in 2010 [41], under field conditions, it was reported that approximately 83.2% of the total eggs of *B. cockerelli* were missing. Despite the lower percentage of egg hatching in *B. tremblayi* compared to *B. cockerelli* under controlled conditions, both species demonstrated comparable maximum survival rates from egg to adult. In the case of *B. tremblayi*, the maximum survival rate from egg to adult at optimum temperatures was recorded as 35.24%, while *B. cockerelli* exhibited values of 37% on potato and between 40 and 42% on tomato [38,40]. 

Field development times from hatching egg to emerging adult in psyllid species can vary depending on factors such as the biology and habitat of the species and temperature conditions, among others [5]. In some non-diapausing tropical/subtropical species, the development times typically range between 9.5 and 23 days [5]. Warm to cool temperate-adapted species generally have development times that span around 22 to 44 days [5]. However, it is worth noting that development can be significantly slower at temperatures just above the developmental threshold, extending their development to 190 days in species like *Trioza urticae* [5]. According to Tran et al. in (2012) [42], *B. cockerelli* completed its entire cycle in 21.11 days on potato and 22.08 days on tomato under optimum temperature conditions. In the controlled environment at 25 °C, *B. tremblayi* displayed its minimum total development period, completing it in 26.72 days. In a comparative study conducted by Kazemi and Jafarloo in 2008 [31], *B. tremblayi* displayed developmental times of 33.63 days for males and 39.94 days for females at 21 °C. These values surpassed the developmental time of 31.61 days recorded at 20 °C, a similar temperature to those examined in our current study.

The optimum temperature values estimated represent the temperature at which the developmental time is shortest, which has been considered by many authors [43,44,45]. It should be noted, however, that this temperature is not necessarily the one that allows insects to develop fastest with low survival and net reproductive rate, a parameter known as fast temperature (Tfast). The calculation of optimal and critical temperatures in most of the models is based on the developmental time, not including the survival rates. In our study, the survival rate of *B. tremblayi* reached its maximum at 20–25 °C. The outcomes suggested by the Briere, Lactin, and Taylor models defined Topt for the development of *B. tremblayi* near 25 °C. Conversely, the results obtained from the SSI model showed Topt’ in closer proximity to 20 °C. The maximum peaks in field conditions were observed when mean daily temperatures showed values close to 15 °C, which implies maximum temperatures of 20–25 °C. Mean temperatures up to 25 °C involve recording maximum temperature peaks of more than 30 °C, the critical temperature where no plague development occurs. Consequently, the range of temperatures for the development of nymphs provided by the models used resulted in a good adjustment in predicting the survival patterns of *B. tremblayi* under the studied environmental conditions.

Our study also found that the temperature did not exert a significant influence on the sex ratio and the duration of development time for males and females. These findings align with the study conducted by Kazemi and Jafarloo in 2008 [31] on *B. tremblayi*, which similarly did not observe significant differences in developmental time by gender. However, it is noteworthy that the proportion of females and males was higher and significantly different in our study under controlled conditions, contrasting with the data obtained from field observations. For *B. tremblayi*, the proportion of males was notably higher than females in both sampling methods used to capture adult psyllids, particularly in the horizontal green water traps, which proved effective in capturing early immigrants, yielding male proportions of approximately 88%. In the context of studies conducted on *T. apicalis* concerning migration, differences in sex ratio were attributed to geographical factors or monitoring methods. Specifically, it was reported that at the onset of the flight period, males are more frequent, while females become more numerous towards the end [46]. Differences in behaviour between genders can also influence adult captures, with increased activity in males, particularly related to mate-searching behaviour, and decreased activity in gravid females [47,48]. However, a higher number of carrot psyllid females could potentially lead to greater damage in plants [49]. This may be attributed to the substantial nutrient requirements of *T. apicalis* females to support the production of a large number of eggs, leading to variations in plant damage correlated with the duration of psyllid feeding [49]. 

## 5. Conclusions

*Bactericera tremblayi* is the prevalent psyllid species encountered within the surveyed leek fields in the cultivation region of Castile and Leon, being identified as the primary cause of damage founded in this crop over the recent growing seasons. The plague development and the population dynamics of this species exhibit a marked susceptibility to temperature influences, with its optimal range falling within the range of 20 to 25 °C. Furthermore, it offers the potential to develop predictive models to anticipate its seasonal occurrence, with a particular focus on climate-related factors as temperature. This knowledge rationalizes the implementation of effective population management strategies for this pest species, thereby contributing to the mitigation of its potential impact on agricultural systems. On the other hand, it is essential to conducting comprehensive research into other potential factors that exert an impact on *B. tremblayi* populations in field conditions, such as the role of natural predators and parasitoids. Such explorations will refine the integrated control management of this pest.

## Figures and Tables

**Table 1:** Location and information of leek fields periodically surveyed during the cultivation cycles of 2017, 2018, 2019, and 2020 in Castile and Leon (Spain).

Year	Field Location	Province	Latitude	Longitude	Management	Crop Cycle ^1^
2017	Chatún	Segovia	41°17′4.59″ N	4°22′4.81″ W	Organic	MS
2017	San Pablo de la Moraleja	Valladolid	41°9′53.90″ N	4°45′10.90″ W	Integrated	MS
2018	Arroyo de Cuéllar	Segovia	41°21′25.89″ N	4°20′38.98″ W	Integrated	LS
2018	Ataquines	Valladolid	41°12′17.30″ N	4°46′36.59″ W	Integrated	LS
2019	Campo de Cuéllar	Segovia	41°18′33.25″ N	4°21′29.41″ W	Organic	LS
2019	Chañe	Segovia	41°19′53.65″ N	4°24′15.37″ W	Integrated	LS
2020	Íscar	Valladolid	41°20′17.38″ N	4°31′32.96″ W	Organic	LS

^1^ Type of crop cycle: mid-season (MS) and late-season (LS).

**Table 2:** Number of captured psyllids of *Bactericera nigricornis* Förster group by species and gender using horizontal green tile water traps (HGWT) and sweep net sampling (SNS) in leek fields periodically surveyed in 2017, 2018, 2019, and 2020 in Castile and Leon (Spain).

Year	Locality	*Bactericera trigonica*				*Bactericera nigricornis*				*Bactericera tremblayi*			
		HGWT		SNS		HGWT		SNS		HGWT		SNS	
		Males	Females	Males	Females	Males	Females	Males	Females	Males	Females	Males	Females
2017	Chatún	6	4	11	1	40	1	1	0	14	1	373	160
	San Pablo de la Moraleja	5	0	3	2	22	4	2	0	27	6	1067	302
2018	Arroyo de Cuéllar	1	0	0	0	0	0	3	8	8	3	3059	620
	Ataquines	0	0	8	2	2	0	1	3	20	2	206	56
2019	Campo de Cuéllar	1	2	6	3	2	0	7	1	68	13	581	202
	Chañe	30	12	1	3	0	0	0	0	113	12	2834	700
2020	Íscar	16	3	0	5	2	1	5	0	10	0	65	67
Total by gender		59	21	29	16	68	6	19	12	260	37	8185	2107
Percentage by gender		73.75	26.25	64.44	35.56	91.89	8.11	61.29	38.71	87.54	12.46	79.53	20.47
Total by species		80		45		74		31		297		10,292	
Percentage by species		17.74		0.43		16.41		0.30		65.85		99.27	

**Table 3:** Developmental time (days) of different stages of *B. tremblayi* at four constant temperatures.

Temperature	Egg	N1	N2	N3	N4	N5
15	16.09 a	26.35 a	32.87 a	42.42 a	46.88 a	51.5 a
20	9.85 b	16.09 b	20.36 b	23.87 b	26.18 b	31.61 b
25	8.18 bc	13.89 b	17.11 b	20.34 b	22.56 b	26.72 b
30	6.19 c	10.67 b	ND	-	-	-
F	72.76	26.55	26.54758	33.674	19.27	36.51
df between groups	3	3	2	2	2	2
df within groups	11	11	8	8	8	8
*p*	6.68 × 10^−6^	0.00043	0.00043	0.0006615	0.00136	0.00052

Means in a column followed by different letters are significantly different (Tukey HSD, *p* < 0.05). ND = Not developed.

**Table 4:** Survival (%) of different stages of *B. tremblayi* at four constant temperatures.

Temperature	Egg	N1	N2	N3	N4	N5	Egg to Adult
15	46.02 a	82.12 a	95.24 a	96.42 a	96.73 a	86.93 a	29.45 a
20	56.39 ab	87.46 a	92.55 a	92.58 a	93.40 a	89.51 a	35.24 a
25	52.60 ab	84.91 a	90.04 a	89.35 a	90.50 a	83.28 a	32.07 a
30	8.45 b	8.69 b	0.00 b	-	-	-	0.00 b
F	5.759	11.9	17.69	19.27	18.48	16.56	5.564
df between groups	3	3	3	2	2	2	3
df within groups	11	11	11	8	8	8	11
*p*	0.0373	0.00623	0.00181	0.00136	0.00157	0.00225	0.04

Means in a column followed by different letters are significantly different (Tukey HSD, *p* < 0.05).

**Table 5:** Estimated parameters of the models fitting developmental rates of *B. tremblayi*.

	Parameter	Egg	N1	N2	N3	N4	N5	Reference
Campbell	Tmin	4.99	−0.21	3.73	5.32	5.13	3.80	[15]
	R-square	0.93	0.91	0.96	0.97	0.95	0.96	
Briere1_99	Tmin	−13.98	0	3.91	7.27	7.6	4.94	[23]
	Topt	46.35	29.24	25.12	24.35	24.11	24.69	
	Tmax	59.45	-	30.87	29.37	29.01	30.18	
	R-square	0.94	0.91	0.90	0.90	0.93	0.95	
Taylor	Topt	41.68	29.40	25.76	24.67	24.34	25.20	[25]
	R-square	0.94	0.95	0.90	0.90	0.93	0.95	
Lactin	Topt	34.24	28.84	24.33	23.84	23.71	24.08	[24]
	Tmax	43.40	37.25	30.04	28.71	28.5	29.57	
	R-square	0.94	0.96	0.90	0.90	0.93	0.95	
SSI	Topt’	20.6	22.15	21.93	20.45	20.34	20.54	[26]
	TL	6.5	8.63	8.80	8.14	7.82	6.57	
	TH	37.18	31.95	25.83	26.72	26.76	26.92	
	R-square	0.94	0.79	0.79	0.87	0.89	0.90	

## Data Availability

Data are contained within the article or Supplementary Material.

## Supplementary Materials

The following supporting information can be downloaded at: https://www.mdpi.com/article/10.3390/insects15010004/s1, Table S1. Location, number of individuals of *B. tremblayi* captured with sweep nets, and percentage of plants with juvenile stages in leek plots subject to extensive survey in Castile and León (Spain) from 2017 to 2019; Figure S1. Number of individuals of *Bactericera tremblayi* captured by sweep (first y-axis) and year in leek plots subject to seasonal monitoring in Castile and Leon (Spain) from 2017 to 2020. Average temperature is shown on the second axis (solid blue line); Figure S2. Number of eggs (red line), nymphs N1–N2 (orange line), and nymphs N3–N5 (green line) observed on plants in leek plots subject to seasonal monitoring in Castile and Leon (Spain) from 2017 to 2020. Scale used 0 = 0, 1 = 1–4. 2 = 5–20, 3 = 21–50, 4 = more than 50 (first y-axis). Average temperature is shown on the second axis (solid blue line).

## References

[1] MAPA Estadísticas Agrarias: Agricultura. https://www.mapa.gob.es/es/estadistica/temas/estadisticas-agrarias/agricultura/default.aspx.

[2] Asensio-S.Manzanera M.C., Santiago-Calvo Y., Ruano-Rosa D., Vacas-Izquierdo R., Flores-Pérez D. Evolución de las poblaciones de *Bactericera tremblayi* (Wagner, 1961) (Insecta: Hemiptera: Sternorrhyncha: Psylloidea) en cultivos hortícolas de Castilla y León y su posible relación con los síntomas aparecidos. Proceedings of the XI Congreso Nacional de Evaluación de Impacto Ambiental.

[3] Ouvrard D., Burckhardt D. (2012). First Record of the Onion Psyllid *Bactericera tremblayi* (Wagner, 1961) in France (Insecta: Hemiptera: Steirnorrhyncha: Psylloidea), New Symptoms on Leek Crops and Reassessment of the *B. nigricornis*-Group Distribution. EPPO Bull..

[4] Hodkinson D. (1981). Status and Taxonomy of the *Trioza (Bactericera) nigricornis* Förster Complex (Hemiptera: Triozidae). Bull. Entomol. Res..

[5] Hodkinson D. (2009). Life Cycle Variation and Adaptation in Jumping Plant Lice (Insecta: Hemiptera: Psylloidea): A Global Synthesis. J. Nat. Hist..

[6] EPPO Bactericera tremblayi (TRIZTE) [World Distribution] EPPO Global Database. https://gd.eppo.int/taxon/TRIZTE/distribution.

[7] Conci C., Rapisarda C., Tamanini L. (1996). Annotated Catalogue of the Italian Psylloidea. Second Part (Insecta Homoptera). Atti della Accad. Roveretana degli Agiati Ser. 7 B. Cl. di Sci. Mat. Fis. e Nat..

[8] Jerinic-Prodanovic D. (2006). Distribution, Biology and Harmfulnes of Jumping Plant-Louse *Bactericera tremblayi* Wagner (Homoptera, Triozidae) in Serbia. Pestic. Phytomedicine.

[9] Teresani G., Hernández E., Bertolini E., Siverio F., Marroquín C., Molina J., de Mendoza A.H., Cambra M. (2015). Search for Potential Vectors of *Candidatus* Liberibacter Solanacearum: Population Dynamics in Host Crops. Spanish J. Agric. Res..

[10] Antolínez C., Moreno A., Ontiveros I., Pla S., Plaza M., Sanjuan S., Palomo J.L., Sjölund M.J., Sumner-Kalkun J., Arnsdorf Y. (2019). Seasonal Abundance of Psyllid Species on Carrots and Potato Crops in Spain. Insects.

[11] Asensio-S.Manzanera M.C., Santiago-Calvo Y., Palomo-Gómez J.L.J.J.L.J., Marquínez-Ramírez R., Bastin S., García-Méndez E.E.M., Hernández-Suárez E., Siverio-de-la-Rosa F., Asensio-S.-Manzanera M.C., Santiago-Calvo Y. (2022). Survey of *Candidatus* Liberibacter Solanacearum and Its Associated Vectors in Potato Crop in Spain. Insects.

[12] Teresani G., Bertolini E., Alfaro-Fernández A., Martínez C., Tanaka F.A.O., Kitajima E.W., Roselló M., Sanjuán S., Ferrándiz J.C., López M.M. (2014). Association of *Candidatus* Liberibacter Solanacearum with a Vegetative Disorder of Celery in Spain and Development of a Real-Time Pcr Method for Its Detection. Phytopathology.

[13] Moreno A., Miranda M.P., Fereres A. (2021). Psyllids as Major Vectors of Plant Pathogens. Entomol. Gen..

[14] Antolínez C., Fereres A., Moreno A. (2017). Risk Assessment of *Candidatus* Liberibacter Solanacearum Transmission by the Psyllids *Bactericera trigonica* and *B. tremblayi* from Apiaceae Crops to Potato. Sci. Rep..

[15] Campbell A., Frazer B.D., Gilbert N.G.A.P., Gutierrez A.P., Mackauer M. (1974). Temperature Requirements of Some Aphids and Their Parasites. J. Appl. Ecol..

[16] Damos P., Savopoulou-Soultani M. (2012). Temperature-Driven Models for Insect Development and Vital Thermal Requirements. Psyche.

[17] Saeidi Z., Nemati A. (2017). Relationship between Temperature and Developmental Rate of *Schizotetranychus smirnovi* (Acari: Tetranychidae) on Almond. Int. J. Acarol..

[18] MAPA Registro de Productos Fitosanitarios. https://servicio.mapa.gob.es/regfiweb.

[19] MAPA Guía de Gestión Integrada de Plagas: Liliaceas. https://www.mapa.gob.es/es/agricultura/temas/sanidad-vegetal/guiagip-liliaceasprotegida_tcm30-434394.pdf.

[20] European Commission Commission Implementing Regulation (EU) 2021/1165 of 15 July 2021 Authorising Certain Products and Substances for Use in Organic Production and Establishing Their Lists (Text with EEA Relevance). https://eur-lex.europa.eu/eli/reg_impl/2021/1165/oj/spa.

[21] Irwin M.E. (1980). Sampling Aphids in Soybean Fields. Sampling Methods in Soybean Entomology.

[22] Irwin M.E., Ruesink W.G., McLean G.D., Garrett R.G., Ruesink W.G. (1986). Vector Intensity: A Product of Propensity and Activity. Plant Virus Epidemics: Monitoring, Modelling and Predicting Outbreaks.

[23] Briere J.F., Pracros P., le Roux A.Y., Pierre S. (1999). A Novel Rate Model of Temperature-Dependent Development for Arthropods. Environ. Entomol..

[24] Lactin D.J., Holliday N.J., Johnson D.L., Craigen R. (1995). Improved Rate Model of Temperature-Dependent Development by Arthropods. Environ. Entomol..

[25] Taylor F. (1981). Ecology and Evolution of Physiological Time in Insects. Am. Nat..

[26] Ikemoto T., Kurahashi I., Shi P.J. (2013). Confidence Interval of Intrinsic Optimum Temperature Estimated Using Thermodynamic SSI Model. Insect Sci..

[27] Kontodimas D., Eliopoulos P., Stathas G., Economou L. (2004). Comparative Temperature-Dependent Development of *Nephus includens* (Kirsch) and *Nephus bisignatus* (Boheman) (Coleoptera: Coccinellidae) Preying on *Planococcus citri* (Risso) (Homoptera: Pseudococcidae): Evaluation of a Linear and Variou. Environ. Entomol..

[28] Ikemoto T. (2005). Intrinsic Optimum Temperature for Development of Insects and Mites. Environ. Entomol..

[29] Ikemoto T. (2008). Tropical Malaria Does Not Mean Hot Environments. J. Med. Entomol..

[30] Shi P., Ikemoto T., Egami C., Sun Y., Ge F. (2011). A Modified Program for Estimating the Parameters of the SSI Model. Environ. Entomol..

[31] Kazemi M.H., Jafarloo M.M. (2008). Laboratory Investigation of the Biology of *Bactericera tremblayi* Wag. (Homoptera: Triozidae) a New Pest in Onion Fields of Iran. Am. J. Agric. Biol. Sci..

[32] Veres A., Petit S., Conord C., Lavigne C. (2013). Does Landscape Composition Affect Pest Abundance and Their Control by Natural Enemies? A Review. Agric. Ecosyst. Environ..

[33] Togni P.H.B., Harterreiten-Souza É.S., Novaes D.R., Sujii E.R. (2021). Spatial Dynamic and Spillover of the Polyphagous Pest *Bemisia tabaci* Is Influenced by Differences in Farmland Habitats on Tropical Organic Farms. Agric. Ecosyst. Environ..

[34] Mazzi D., Dorn S. (2012). Movement of Insect Pests in Agricultural Landscapes. Ann. Appl. Biol..

[35] Cranshaw W.S., Zehnder G.W., Powelson M.L., Janson R.K., Raman K.V. (1994). The Potato (Tomato) Psyllid, Paratrioza cockerelli (Sulc), as a Pest of Potatoes. Advances in Potato Pest Biology and Management.

[36] Láska P. (2013). Migration Flight of Carrot Psyllid (*Trioza apicalis*) at Various Latitudes Is Independent of Local Phenology. Plant Prot. Sci..

[37] Djaman K., Higgins C., Begay S., Koudahe K., Allen S., Lombard K., O’Neill M. (2019). Seasonal Occurrence of Potato Psyllid (*Bactericera cockerelli*) and Risk of Zebra Chip Pathogen (*Candidatus* Liberibacter Solanacearum) in Northwestern New Mexico. Insects.

[38] Tran L.T., Worner S.P., Hale R.J., Teulon D.A.J. (2012). Estimating Development Rate and Thermal Requirements of *Bactericera cockerelli* (Hemiptera: Triozidae) Reared on Potato and Tomato by Using Linear and Nonlinear Models. Environ. Entomol..

[39] Yang X.B., Liu T.X. (2009). Life History and Life Tables of *Bactericera cockerelli* (Homoptera: Psyllidae) on Eggplant and Bell Pepper. Environ. Entomol..

[40] Abdullah N. (2008). Life History of the Potato Psyllid *Bactericera cockerelli* (Homoptera: Psyllidae) in Controlled Environment Agriculture in Arizona. African J. Agric. Res..

[41] Yang X.B., Zhang Y.M., Hua L., Peng L.N., Munyaneza J.E., Trumble J.T., Liu T.X. (2010). Repellency of Selected Biorational Insecticides to Potato Psyllid, *Bactericera cockerelli* (Hemiptera: Psyllidae). Crop Prot..

[42] Tran L. (2012). Population Phenology, Life Table and Forecasting Models of Tomato-Potato Psyllid (*Bactericera cockerelli*) and the Efficiency of a Selected Natural Enemy for Its Control. Ph.D. Thesis.

[43] Moallem Z., Karimi-Malati A., Sahragard A., Zibaee A. (2017). Modeling Temperature-Dependent Development of *Glyphodes pyloalis* (Lepidoptera: Pyralidae). J. Insect Sci..

[44] Aghdam H.R., Fathipour Y., Radjabi G., Rezapanah M. (2009). Temperature-Dependent Development and Temperature Thresholds of Codling Moth (Lepidoptera: Tortricidae) in Iran. Environ. Entomol..

[45] Zahiri B., Fathipour Y., Khanjani M., Moharramipour S., Zalucki M.P. (2010). Preimaginal Development Response to Constant Temperatures in *Hypera postica* (Coleoptera: Curculionidae): Picking the Best Model. Environ. Entomol..

[46] Láska P. (2011). Biology of *Trioza apicalis*—A Review. Plant Prot. Sci..

[47] Blackmer J.L., Cañas L.A. (2005). Visual Cues Enhance the Response of *Lygus hesperus* (Heteroptera: Miridae) to Volatiles from Host Plants. Environ. Entomol..

[48] Nissinen A.I., Kristoffersen L., Anderbrant O. (2008). Physiological State of Female and Light Intensity Affect the Host-Plant Selection of Carrot Psyllid, *Trioza apicalis* (Hemiptera: Triozidae). Eur. J. Entomol..

[49] Nissinen A.I., Haapalainen M., Jauhiainen L., Lindman M., Pirhonen M. (2014). Different Symptoms in Carrots Caused by Male and Female Carrot Psyllid Feeding and Infection by *Candidatus* Liberibacter Solanacearum. Plant Pathol..

