# Supplementary material for: Bactericera tremblayi (Wagner, 1961) (Hemiptera: Triozidae): The Prevalent Psyllid Species in Leek Fields of Northwestern Spain
Source: Insects. 2023 Dec 21;15(1):4. doi: 10.3390/insects15010004 (PMC10816366; doi:10.3390/insects15010004)
Supplement: Supplementary file 1 [file insects-15-00004-s001.zip › Figure S1.pdf]

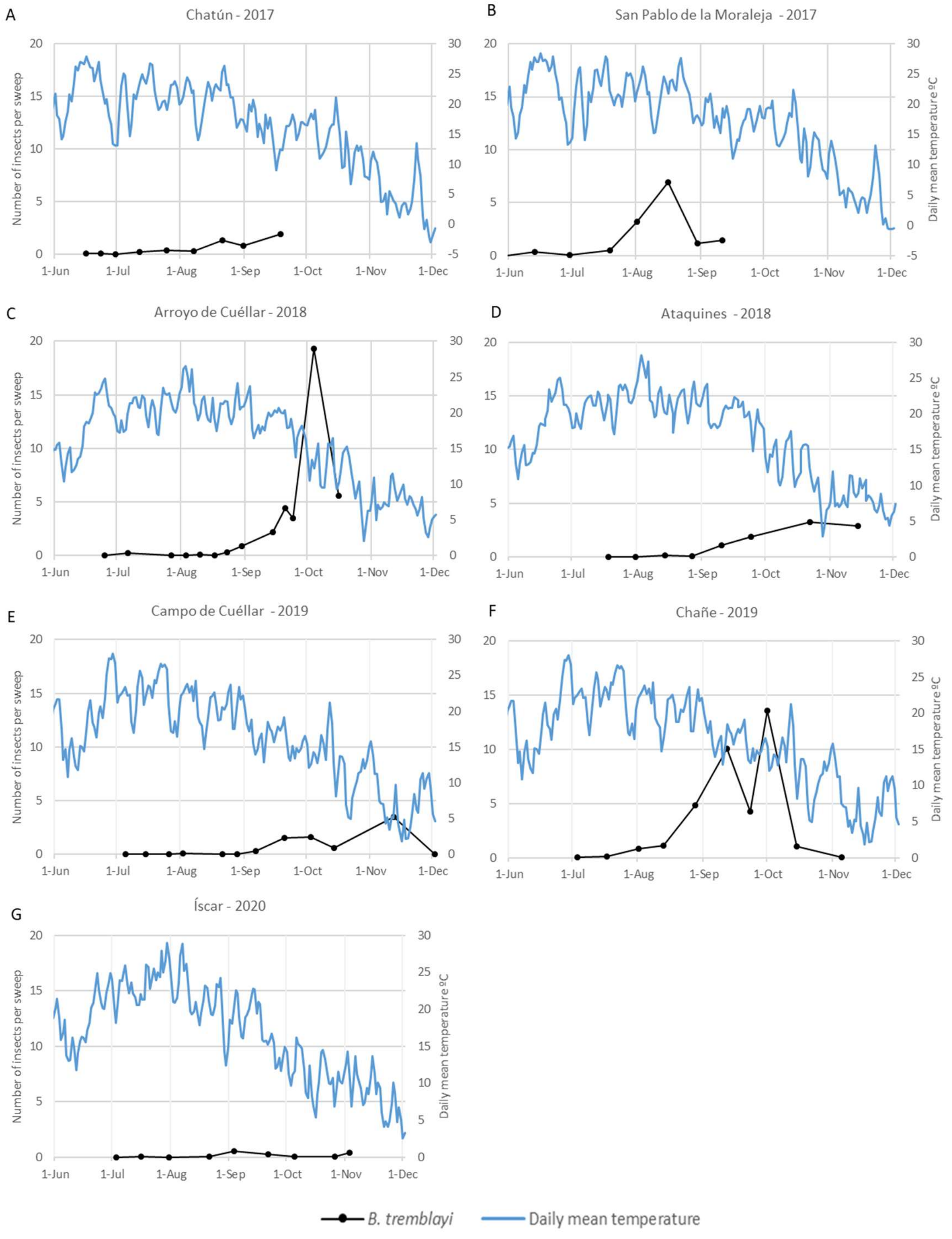

Figure S1. Number of individuals of *Bactericera tremblayi* captured by sweep (first y-axis) and year in leek plots subject to seasonal monitoring in Castile and Leon (Spain) from 2017 to 2020. Average temperature is shown on the second axis (solid blue line)
