# Supplementary material for: Bactericera tremblayi (Wagner, 1961) (Hemiptera: Triozidae): The Prevalent Psyllid Species in Leek Fields of Northwestern Spain
Source: Insects. 2023 Dec 21;15(1):4. doi: 10.3390/insects15010004 (PMC10816366; doi:10.3390/insects15010004)
Supplement: Supplementary file 1 [file insects-15-00004-s001.zip › Figure S2.pdf]

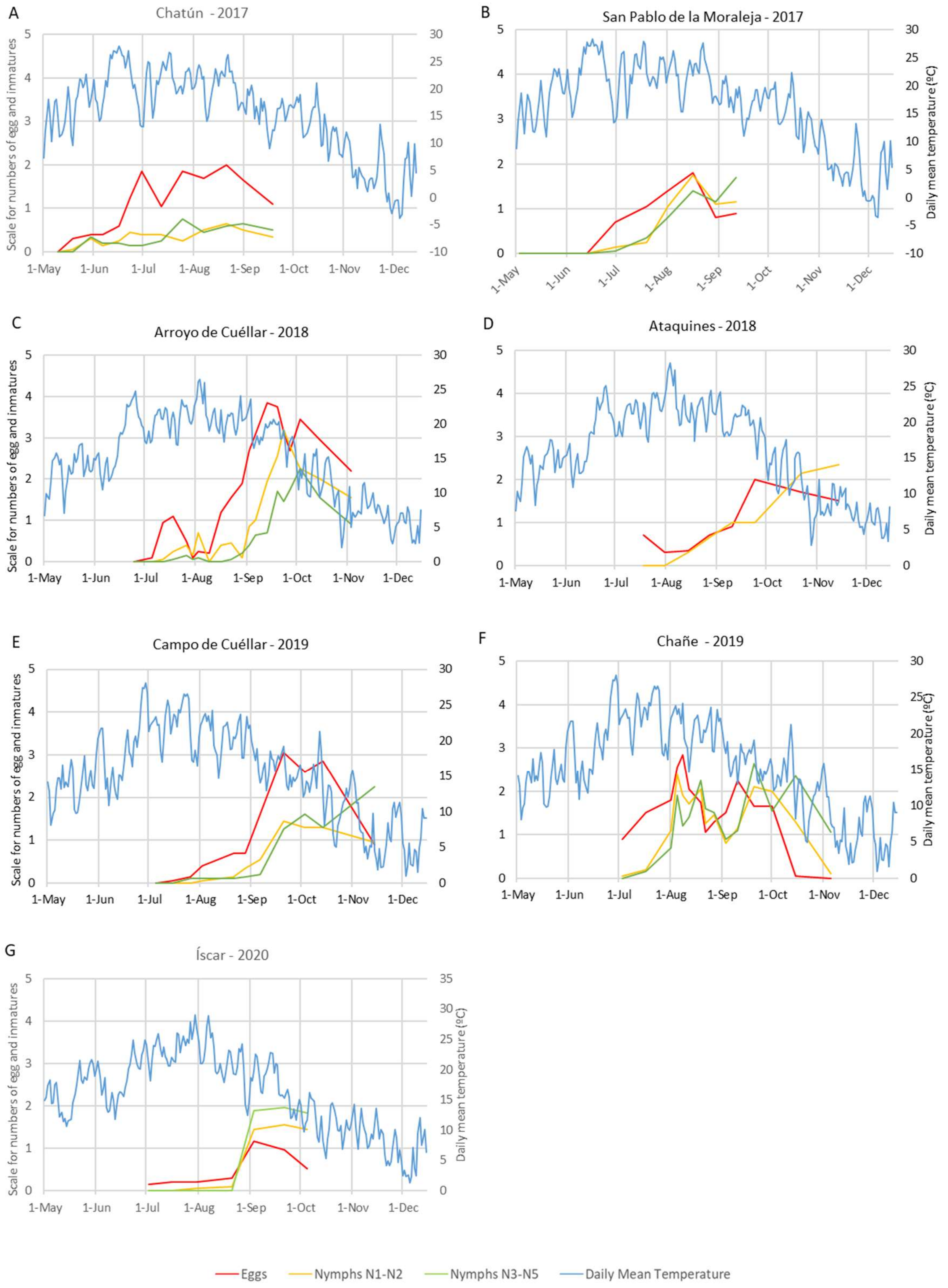

Figure S2. Number of eggs (red line), nymphs N1–N2 (orange line), and nymphs N3–N5 (green line) observed on plants in leek plots subject to seasonal monitoring in Castile and Leon (Spain) from 2017 to 2020. Scale used 0 = 0, 1 = 1–4. 2 = 5–20, 3 = 21–50, 4 = more than 50 (first y-axis). Average temperature is shown on the second axis (solid blue line).
