# Supplementary material for: Bactericera tremblayi (Wagner, 1961) (Hemiptera: Triozidae): The Prevalent Psyllid Species in Leek Fields of Northwestern Spain
Source: Insects. 2023 Dec 21;15(1):4. doi: 10.3390/insects15010004 (PMC10816366; doi:10.3390/insects15010004)
Supplement: Supplementary file 1 [file insects-15-00004-s001.zip › Table S1.pdf]

Table S1. Location, number of individuals of *B. tremblayi* captured with sweep nets, and percentage of plants with juvenile stages in leek plots subject to extensive survey in Castile and León (Spain) from 2017 to 2019.

Locality	Province	Type of crop cycle[1]	Crop development	Date	insect/sweep	(%) Plants with inmature forms
Chatún	Segovia	MS	Beginning	29/05/2017	0.06	10
Villagonzalo de Coca	Segovia	MS	Beginning	05/06/2017	0.12	18
Alcazarén	Valladolid	MS	Beginning	16/06/2017	0.52	70
Navalmanzano	Segovia	MS	Beginning	22/06/2017	0.06	42
Vinaderos	Ávila	MS	Beginning	04/07/2017	0.00	22
Mozoncillo	Segovia	MS	Beginning	17/07/2017	.	42
Hornillos de Eresma	Valladolid	MS	Beginning	27/07/2017	0.00	18
Íscar	Valladolid	MS	Beginning	27/07/2017	0.34	42
Campo de Cuéllar	Segovia	MS	Beginning	04/06/2018	0.06	42
Bóveda de Toro	Zamora	MS	Beginning	04/07/2018	0.08	2
San Martín y Mudrián	Segovia	LS	Beginning	21/07/2017	0.00	16
Arroyo de Cuéllar	Segovia	LS	Beginning	22/07/2017	0.02	32
Villeguillo	Segovia	LS	Beginning	25/07/2017	0.00	0
Tordesillas	Valladolid	LS	Beginning	28/07/2017	0.00	20
Villanueva de Duero	Valladolid	LS	Beginning	28/07/2017	2.32	100
Armuña	Segovia	LS	Beginning	03/08/2018	0.02	14
Ataquines	Valladolid	LS	Beginning	14/08/2018	0.18	64
Villeguillo	Segovia	LS	Beginning	27/06/2019	0.06	0
Chañe	Segovia	LS	Beginning	17/07/2019	0.14	100
Campo de Cuéllar	Segovia	LS	Beginning	26/07/2019	0.02	24
Hornillos de Eresma	Valladolid	LS	Beginning	31/07/2019	0.08	20
Chatún	Segovia	LS	Beginning	22/08/2019	0.14	66
Villagonzalo de Coca	Segovia	MS	Middle crop cycle	23/07/2017	0.52	0
Navalmanzano	Segovia	MS	Middle crop cycle	25/07/2017	0.04	30
Chatún	Segovia	MS	Middle crop cycle	27/07/2017	0.14	32
Alcazarén	Valladolid	MS	Middle crop cycle	11/08/2017	0.06	86
Vinaderos	Ávila	MS	Middle crop cycle	31/08/2017	0.04	2
Mozoncillo	Segovia	MS	Middle crop cycle	01/09/2017	0.20	64
Hornillos de Eresmá	Valladolid	MS	Middle crop cycle	25/09/2017	0.12	26
Íscar	Valladolid	MS	Middle crop cycle	26/09/2017	1.86	58
Campo de Cuéllar	Segovia	MS	Middle crop cycle	24/07/2018	0.22	88
Bóveda de Toro	Zamora	MS	Middle crop cycle	23/08/2018	0.14	16
Arroyo de Cuéllar	Segovia	LS	Middle crop cycle	30/08/2017	0.12	82
San Martín y Mudrián	Segovia	LS	Middle crop cycle	01/09/2017	.	56
Tordesillas	Valladolid	LS	Middle crop cycle	05/09/2017	0.30	36
Villanueva de Duero	Valladolid	LS	Middle crop cycle	05/09/2017	0.32	100
Villeguillo	Segovia	LS	Middle crop cycle	06/09/2017	0.20	10
Armuña	Segovia	LS	Middle crop cycle	23/08/2018	0.04	12
Ataquines	Valladolid	LS	Middle crop cycle	10/09/2018	1.44	70
Campo de Cuéllar	Segovia	LS	Middle crop cycle	21/08/2019	6.88	100
Chañe	Segovia	LS	Middle crop cycle	28/08/2019	2.04	100
Chatún	Segovia	LS	Middle crop cycle	20/09/2019	2.04	100
Hornillos de Eresma	Valladolid	LS	Middle crop cycle	23/09/2019	0.36	98
Villeguillo	Segovia	LS	Middle crop cycle	24/09/2019	0.46	42
Navalmanzano	Segovia	MS	Harvest	23/08/2017	0.32	50
Villagonzalo de Coca	Segovia	MS	Harvest	06/09/2017	0.08	20
Chatún	Segovia	MS	Harvest	17/09/2017	0.52	20
Vinaderos	Ávila	MS	Harvest	18/09/2017	0.00	0
Alcazarén	Valladolid	MS	Harvest	17/10/2017	0.98	76
Bóveda de Toro	Zamora	MS	Harvest	10/10/2018	0.12	96
Campo de Cuéllar	Segovia	MS	Harvest	18/10/2018	4.16	100
Arroyo de Cuéllar	Segovia	LS	Harvest	01/10/2017	1.76	100
Villeguillo	Segovia	LS	Harvest	09/10/2017	0.56	56
Tordesillas	Valladolid	LS	Harvest	26/10/2017	7.98	96
San Martín y Mudrián	Segovia	LS	Harvest	22/11/2017	1.14	42
Armuña	Segovia	LS	Harvest	18/10/2018	0.84	100

Arroyo de Cuéllar	Segovia	LS	Harvest	23/10/2018	4.86	100
Hornillos de Eresma	Valladolid	LS	Harvest	29/10/2018	2.02	100
Gomezserracín	Segovia	LS	Harvest	13/11/2018	0.56	100
Gomezserracín	Segovia	LS	Harvest	13/11/2018	1.1	76
Ataquines	Valladolid	LS	Harvest	14/11/2018	2.78	86
Gomezserracín	Segovia	LS	Harvest	07/02/2019	0.22	46
Villeguillo	Segovia	LS	Harvest	07/02/2019	1.02	60
Campo de Cuéllar	Segovia	LS	Harvest	23/09/2019	4.92	100
Chañe	Segovia	LS	Harvest	09/10/2019	3.80	100
Hornillos de Eresma	Valladolid	LS	Harvest	15/10/2019	0.74	100
Chatún	Segovia	LS	Harvest	28/10/2019	16.26	100
Villeguillo	Segovia	LS	Harvest	25/11/2019	0.42	88

[1] Type of crop cycle: mid-season (MS) and late season (LS)
